# miR-146a Ameliorates Liver Ischemia/Reperfusion Injury by Suppressing IRAK1 and TRAF6

**DOI:** 10.1371/journal.pone.0101530

**Published:** 2014-07-02

**Authors:** Weiwei Jiang, Liangliang Kong, Qingfeng Ni, Yeting Lu, Wenzhou Ding, Guoqing Liu, Liyong Pu, Weibing Tang, Lianbao Kong

**Affiliations:** 1 Department of Neonatal Surgery, Nanjing Children’s Hospital Affiliated to Nanjing Medical University, Nanjing, China; 2 Department of Liver Transplantation Center, The First Affiliated Hospital of Nanjing Medical University, Nanjing, China; UAE University, Faculty of Medicine & Health Sciences, United Arab Emirates

## Abstract

A critical role of the Toll-like receptor(TLR) and its downstream molecules, including IL-1 receptor-associated kinase 1(IRAK1) and tumor necrosis factor receptor– associated factor 6(TRAF6), in the pathogenesis of liver ischemia/reperfusion (I/R) injury has been documented. Recently a microRNA, miR-146a, was identified as a potent negative regulator of the TLR signaling pathway. In this study, we investigated the role of miR-146a to attenuate TLR signaling and liver I/R injury *in* *vivo* and *in* *vitro*. miR-146a was decreased in mice Kupffer cells following hepatic I/R, whereas IRAK1 and TRAF6 increased. Overexpression of miR-146a directly decreased IRAK1 and TRAF6 expression and attenuated the release of proinflammatory cytokines through the inactivation of NF-κB P65 in hypoxia/reoxygenation (H/R)-induced macrophages, RAW264.7 cells. Knockdown experiments demonstrated that IRAK1 and TRAF6 are two potential targets for reducing the release of proinflammatory cytokines. Moreover, co-culture assays indicated that miR-146a decreases the apoptosis of hepatocytes after H/R. *In vivo* administration of Ago-miR-146a, a stable version of miR-146a *in* *vivo,* protected against liver injury in mice after I/R via inactivation of the TLR signaling pathway. We conclude that miR-146a ameliorates liver ischemia/reperfusion injury *in* *vivo* and hypoxia/reoxygenation injury *in* *vitro* by directly suppressing IRAK1 and TRAF6.

## Introduction

Living-donor liver transplantation (LDLT) is a feasible and valuable surgery for solving the problem of organ shortage. However, many problems are still unsolved, such as ischemia-reperfusion (I/R) injury [1], [2], portal hypertension [3], inflammatory responses [4], and graft rejection [5]. Ischemia-reperfusion (I/R) injury is an inevitable process during liver transplantation, and includes warm and cold ischemia. Activation of intrahepatic immune cells (especially the resident macrophages of the liver, Kupffer cells (KCs)) and oxidative stress in the early phase of warm liver I/R lead to neutrophil accumulation and hepatocellular injury [6]. However, the underlying mechanisms of the exaggerated innate immune response during liver I/R injury remain incompletely defined.

Recently, Toll-like receptors (TLRs), one group of important innate immune receptors, have received much attention. A critical role of the Toll-like receptor 4 (TLR-4) signaling pathway in the pathogenesis of I/R injury has been documented [7]. Activation of TLR4 initiates the transmembrane signaling cascade and triggers intracellular signaling molecules including IL-1 receptor-associated kinase 1 and 4 (IRAK1 and IRAK4) and tumor necrosis factor receptor–associated factor 6 (TRAF6). These signaling molecules induce nuclear translocation of NF-κB and activator protein (AP)-1, resulting in the production of inflammatory cytokines such as TNF-α and IL-6 [8], [9]. Previous studies have also reported that the downregulation of IRAK1 or TRAF6 affects ischemia/reperfusion injury [10], [11].

MicroRNAs (miRNAs), a family of highly and evolutionarily conserved small endogenous non-coding RNA molecules, are silence target mRNA by binding to the 3′UTR of mRNA [12]. Various miRNAs are considered regulators of the innate immune response. A microRNA, miR-146a, was especially identified as a negative regulator of the TLR4 signaling pathway via targeting IRAK1 and TRAF6 in the human acute monocytic leukemia cell line, THP-1 [13].

Besides our previous study [14], no other data are available regarding the role of miR-146a in a warm segmental hepatic I/R model *in* *vivo* and hypoxia/reoxygenation (H/R) model *in* *vitro*. Furthermore, the cell-specific role of miR-146a in a murine hepatic I/R model remains unknown. In the current study, we explored the expression of miR-146a, IRAK1, and TRAF6 in liver I/R Injury. We also revealed the downregulation of TRAF6 and IRAK1 by miR-146a in macrophages following H/R, and found that miR-146a reduces inflammatory responses in macrophages. Taken together, our results show that miR-146a may attenuate the TLR signaling pathway during liver I/R injury.

## Materials and Methods

### Animals

Male C57BL/6J mice (6–8 weeks old) were obtained from our Laboratory Animal Center and fed a standard rodent diet and water in a controlled environment with 12 h light-dark cycles. Mice were fasted for 12 h before the experiment but allowed free access to water. Animals were treated as recommended in the Guide for the Care and Use of Laboratory Animals issued by the Chinese Association for Laboratory Animal Care. All animals experiments were approved by the Nanjing Medical University Laboratory Animal Care and Use Committee.

### Cell culture

The mouse macrophage cell line RAW264.7 was cultured in high-glucose DMEM (Invitrogen, CA, USA) supplemented with 10% fetal bovine serum (FBS, Invitrogen, CA, USA), penicillin (100 units/ml), and streptomycin (100 µg/ml). The mouse hepatocyte cell line AML-12 was maintained in DMEM/F12 medium (Invitrogen, CA, USA) containing 10% FBS, 1×insulin–transferrin–sodium selenite (ITS, Sigma, MO, USA), dexamethasone (40 ng/ml, Sigma, MO, USA), penicillin/streptomycin. All cells were incubated at 37°C in a humidified atmosphere with 5% CO2.

### Liver ischemia/reperfusion (I/R) model

The established non-lethal model of segmental (70%) hepatic warm ischemia/reperfusion was performed as previously described [15]. Briefly, under anesthetization by ether inhalation, the artery and portal venous blood supply to the left and middle liver lobes were interrupted with an atraumatic clip, after a midline laparotomy, for 60 min and the clamp was then removed. Body temperature was maintained at 37°C by handling on a warming pad and heat lamp. Sham animals underwent anesthesia, laparotomy, and exposure of the portal triad without hepatic ischemia. At the end of the predetermined period following reperfusion, the mice were ether inhalation -anesthetized and euthanized by cervical dislocation for tissue and plasma collection.

### Hypoxia/reoxygenation (H/R) model

For exposure to H/R, culture dishes were placed in a chamber with the AnaeroPack-Anaero (MGC, Tokyo, Japan) working as oxygen absorber-CO2 generator according to the manufacturer’s instructions. The oxygen indicator was used to detect oxygen concentration and turned pink when the O_2_ concentration was less than 5%. The chamber was then placed in a cell culture incubator for 1 h or 3 h and reoxygenation achieved by removing the plates from the hypoxic chamber and placing them in a normoxic, humidified incubator for various periods. All experiments (both normoxic and hypoxic) were performed at 37°C.

### Kupffer cells and hepatocytes isolation

Kupffer cells and hepatocytes were isolated from the same mouse liver as previously described [16]. Briefly, after cannulation of the portal vein, the liver was perfused with calcium-free Hank’s balanced salt solution (HBSS, Sigma, MO, USA) at 37°C. Next, the liver was perfused with a 0.05% collagenase IX solution (Sigma, MO, USA) for 5 min. The hepatocytes were filtered, centrifuged under low speed, and washed several times. The viability was assessed using 4% trypan blue (Invitrogen, CA, USA) and was around 85%. Hepatocytes were cultured in DMEM/F12 media supplemented with 10% FBS, 1×ITS, dexamethasone/penicillin/streptomycin. The nonparenchymal cell (NPC) pellet was then resuspended in HBSS, layered onto 25% and 50% Percoll gradients (GE Healthcare, NJ, USA), and centrifuged at 2160×g for 20 min at 4°C. The cells at the interface were washed twice, resuspended, and the viability of the isolated KCs determined by trypan blue, which was usually >90%. Cells were plated in a 12-well culture plate for 2 h at 37°C and then the nonadherent cells were removed. The adherent KCs were cultured in RPMI 1640 medium (Invitrogen, CA, USA) containing 10% FBS and penicillin/streptomycin.

### Real-time RT-PCR

Total RNA was isolated from tissue and cultured cells with the TRIzol reagent (Invitrogen, CA, USA) and reverse-transcribed with a reaction mixture. Quantitative real-time PCR (qPCR) analysis was performed using the SYBR Green qPCR Master Mix (Applied Biosystems, ABI, CA, USA) and a StepOnePlus real-time PCR system (ABI, CA, USA). The expression data were normalized to the expression of β-actin. To determine the miRNA expression, total RNA was reverse-transcribed and the resulting cDNA used with miRNA-specific TaqMan primers (ABI, CA, USA) and TaqMan Universal PCR Master Mix (ABI, CA, USA). RNU6B was used as an endogenous control for data normalization of miRNA levels. The primers used for SYBR Green qPCR or miRNA qPCR are shown in **Table S1**. The comparative threshold cycle (Ct) method was used to measure the relative changes in expression; 2^−ΔΔCt^ represents the fold change of expression, as previously described [17].

### Cell transfection

RAW264.7 cells were cultured in a 6-well culture plate at a density of 2–4×10^5^ cells per well for 24 h, the cells then transfected with the 50 nM miR-146a mimic, miR-146a inhibitor, relative controls, or FAM-negative control miRNA (GenaPharma, Shanghai, China) using Lipofectamine 2000 (Invitrogen, CA, USA) according to the manufacturer’s instructions for 24 h (**Table S2**). To knockdown the expression of TRAK1 or TRAF6, 100 nM IRAK1 small interference RNA (siRNA), TRAF6 siRNA, or negative control siRNA (GenaPharma, Shanghai, China) were transfected for 24 or 48 h (**Table S2**). Then 1 µg/ml LPS or hypoxia/reoxygenation exposure was performed. Each measurement was performed at least three times.

### Dual-luciferase reporter assays

For the 3′UTR luciferase reporter assay, the pmirGLO Dual-Luciferase miRNA Target Expression Vector (Promega, WI, USA) was used. The oligonucleotide sequences (wild and mutated type) used are shown in **Table S3**. The oligonucleotides were ligated into the NheI–XhoI site of pmirGLO. The RAW264.7 cells were co-transfected with the miR-146a mimic (or its control) and constructed pmirGLO vectors and pRL-TK (Promega, WI, USA) using Lipofectamine 2000 (Invitrogen, CA, USA) according to the manufacturer’s instructions for 24 h. For the NF-κB activity assay, RAW264.7 cells were transfected with 1 ug NF-κB luciferase reporter plasmid (Beyotime, Nantong, China), 50 ng pRL-TK, and 50 nM miR-146a mimic or its control for 24 h and then cells were stimulated with 1 µg/ml LPS or H/R. Firefly luciferase and Renilla luciferase luminescence was measured using the Dual-Glo luciferase Reporter Assay System (Promega, WI, USA) and a GloMax 20/20 Luminometer (Promega, WI, USA). Ratios of Firefly luciferase luminescence relative to Renilla luciferase luminescence were calculated.

### ELISA

To determine the release of inflammatory cytokines, cell supernatants were removed and levels of IL-6 and TNF-α were measured using the Valukine ELISA kit (R&D Systems, MN, USA) according to the manufacturer’s instructions.

### Western blot

Total protein from mice livers or cells were obtained with lysis buffer (Beyotime, Nantong, China), resolved by SDS-polyacrylamide gel electrophoresis (PAGE), and transferred to an Immobilon-P polyvinylidene difluoride (PVDF, Millipore, MA, USA) membrane. After blocking the membranes using 5% skim milk for 60 min, we incubated the membranes with an anti-IRAK1 antibody (Abcam, Cambs, UK), anti-TRAF6 antibody (CST, MA, USA), anti-β-ACTIN antibody (Santa Cruz, CA, USA), phosphorylated and total JNK (Santa Cruz, CA, USA). After washing the membranes, we probed with the corresponding secondary antibodies before developing them using an ECL Western blotting detection system (Pierce, NJ, USA) by enhanced chemiluminescence.

### Electrophoretic mobility shift assay

Nuclear extracts from RAW 264.7 cells were performed using a LightShift Chemiluminescent EMSA Kit and a Chemiluminescent Nucleic Acid Detection Module (Pierce, NJ, USA) according to the manufacturer’s instructions. The NF-κB oligonucleotide (5′-AGT TGA GGG GAC TTT CCC AGG C-3′; 3′-TCA ACT CCC CTG AAA GGG TCC G-5′) was labeled with biotin. The equivalent nuclear extract was incubated in gel shift binding buffer for 20 min at room temperature, and the mixtures resolved by 5.5% polyacrylamide gel electrophoresis at 180 V for 45 min and subjected to autoradiography.

### ROS measurement

About 5×10^5^ AML12 cells were stimulated with 0, 10, 20, 40 ng/ml TNF-α (Peprotech, NJ, USA) for 6 h. To determine the ROS level, treated cells were washed with serum-free DMEM/F12 culture medium and incubated with 5 µM dihydroethidium (DHE, Beyotime, Nantong, China) at 37°C for 30 min. Then images were observed using a fluorescent inverted microscope (Nikon, Tokyo, Japan).

### Serum levels of aminopherase

The serum levels of ALT and AST are indexes of hepatocellular injury. A standard automatic analyzer (Hitachi 7600-10, Hitachi, Japan) was used to determine the serum levels of ALT and AST.

### Histology, immunohistochemistry, and TUNEL assay

Formalin-fixed and paraffin-embedded mice liver specimens were sectioned at 4 µm, stained with hematoxylin & eosin, and used for histopathological examinations. Grading of hepatic injury was evaluated using Suzuki’s criteria on a scale from 0 to 4 [18]. For immunohistochemistry staining, the sections were dewaxed and dehydrated. After antigen retrieval in citrate buffer, we blocked the sections overnight at 4°C. The sections were probed with anti-P65 antibody (CST, MA, USA) and an ultra-sensitive immunohistochemistry kit (Maixin, Fuzhou, China) was used. The percentage of P65 positive nuclear was assessed by three independent observers and they were scored as: 0(<5%), 1 (5–25%), 2(26–50%), 3(51–75%), 4(>75%) [19]. Apoptosis of hepatocytes was determined with an *in* *situ* cell death detection kit (Roche, BW, Germany) based on TUNEL (terminal deoxynucleotidyl transferase-mediated dUTP nick end labeling) in liver sections. The sections were observed using light microscopy.

### Co-culture experiments and apoptosis analysis

RAW264.7 cells or KCs were cultured on a 0.4 µm Trans-well membrane insert (Millipore, MA, USA), and AML12 cells or primary hepatocytes were cultured in a 6-well plate. After normoxia (12 h) or H/R (3 h/8 h), the hepatocytes were obtained to determine cell apoptosis using the Annexin-V-FITC Apoptosis Detection Kit (KeyGEN, Nanjing, China) according to the manufacturer’s instruction. Early apoptotic cells were defined as Annexin-V-positive, PI-negative cells. Analyses were performed on a Beckman Gallios Flow Cytometer (Beckman Coulter, CA, USA). Each measurement was repeated three times.

### Therapy of Ago-miR-146a in mice

Male C57BL/6J mice (6–8 weeks of age) were treated with Ago-miR-146a (5 mg/kg) (Ribobio, Guangzhou, China) in saline, its control, or saline alone by tail vein injection as previously described [20]. After 24 h, 60 min of ischemia and 8 h of reperfusion were performed.

### Statistical analysis

Statistical analysis was performed using SPSS software, version 12.0 (SPSS Inc, Ill, USA). Results are expressed as mean values and standard deviations. Parameters were analyzed by Student’s t-test. For the above parameters, P<0.05 was considered statistically significant.

## Results

### miR-146a, IRAK1, and TRAF6 are Expressed During Liver I/R Injury

**Figure 1:**
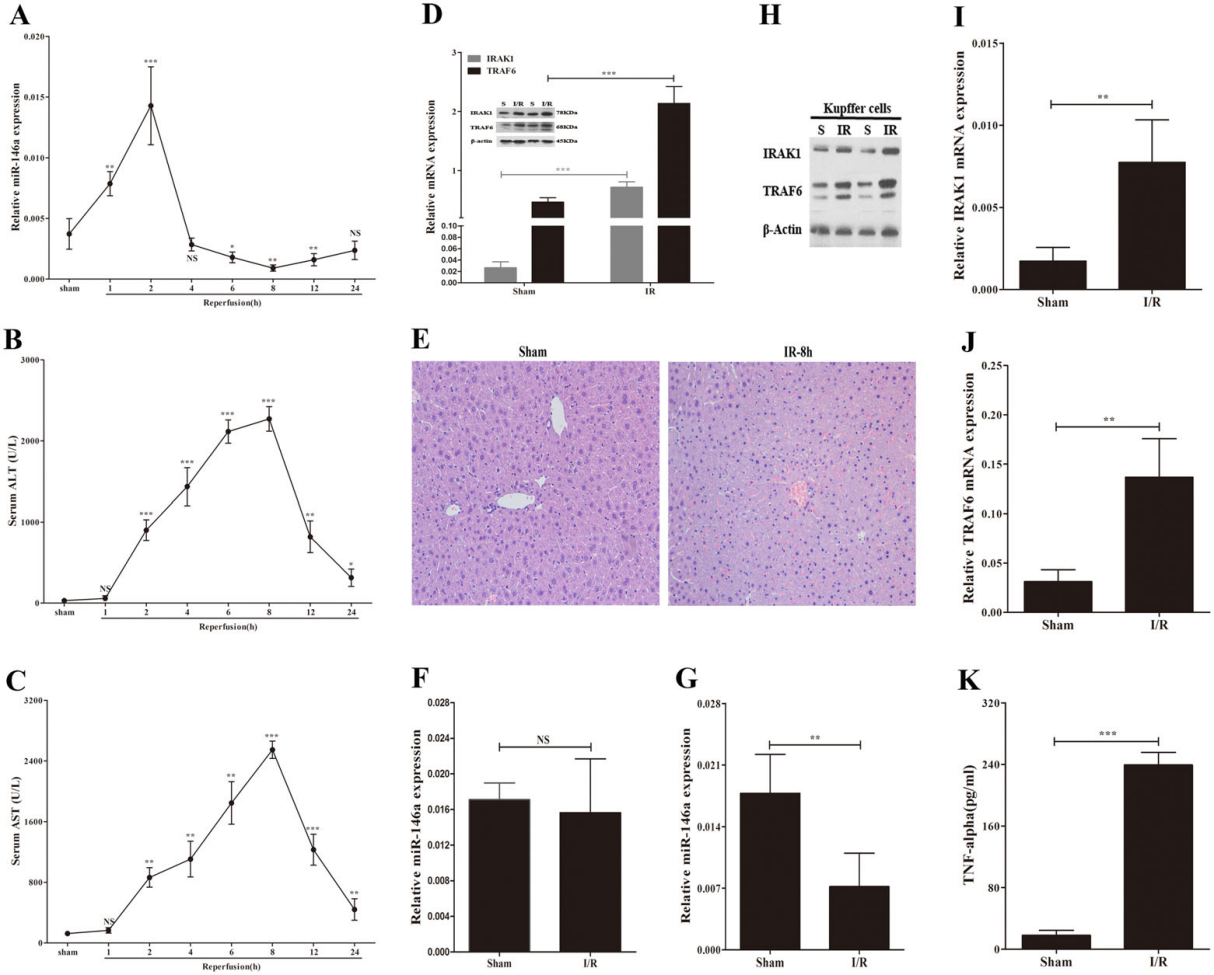
The expression of miR-146a, IRAK1 and TRAF6 during liver ischemia/reperfusion injury. Expression of miR-146a (A) and serum ALT/AST (B and C) release after 60 min of ischemia and various period of reperfusion (1, 2, 4, 6, 8, 12, 24 h) (n = 4–5 mice at each time point). P value vs sham group. (D) RT-PCR analysis and immunoblotting of IRAK1 and TRAF6 in sham group and ischemia/reperfusion (I/R) group (60 min of ischemia and 8 h of reperfusion).(n = 6 mice per group). (E) Representive H&E staning (magnification ×200). Expression of miR-146a (F) in primary hepatocytes, and the levels of miR-146a (G) in Kupffer cells (KCs) isolated from mice following sham operation or I/R (n = 5–6 mice per group). (H) The protein levels of IRAK1 and TRAF6 in KCs. The mRNA levels of IRAK1 (I) and TRAF6 (J) in KCs. (K) Supernatants from KCs were collected to measure TNF-α. Results are mean ± SD. *, P<0.05 and **,P<0.01, ***,P<0.001 versus sham group. S, sham; I/R, ischemia/reperfusion.

We first examined the levels of miR-146a in the murine liver following a sham operation and 60 min of ischemia with various reperfusion times (1, 2, 4, 6, 8, 12, and 24 h). As shown in **Fig. 1A**, miR-146a levels immediately increased in the early phase of reperfusion; however, after 2 hours of reperfusion, miR-146a expression decreased and remained at low levels up to 24 h following reperfusion.

To test the necrosis of hepatocytes, time-dependent levels of serum AST and ALT release following I/R were measured (**Fig. 1B and C**). Thus, we selected 60 min of ischemia and 8 h of reperfusion as a point to evaluate the relationship between miR-146a and key kinases in the TLR signaling pathway, IRAK1 and TRAF6, in the murine liver during I/R injury. The level of miR-146a decreased after I/R, similar to Fig. 1A (data not shown). In contrast, IRAK1 and TRAF6 expression were increased compared with the sham operation (**Fig. 1D**). The H&E assay revealed large areas of hepatocyte necrosis in murine livers subjected to I/R (**Fig. 1E**). These data suggest that IRAK1 and TRAF6 expression is increased in mice following hepatic I/R, whereas the level of miR-146a is decreased.

The activation of TLR signaling in macrophages, including Kupffer cells, plays an important role in hepatic I/R [6]. One of the most prominent miRNAs regulating TLR signaling pathway in macrophages is miR-146a [21]. We therefore determined whether KCs or hepatocytes had decreased levels of miR-146a in mice following I/R. Levels of miR-146a, TRAF6, and IRAK1 in KCs or hepatocytes, gradiently separated from murine livers after the sham operation or hepatic I/R, were determined by RT-PCR analysis. Liver I/R led to the reduction of miR-146a expression and the upregulation of TRAF6 and IRAK1 expression (**Fig. 1G–J**) in KCs. However, the levels of miR-146a remained unchanged in hepatocytes compared with the sham operation (**Fig. 1F**). Meanwhile, we found that I/R induced a significantly increased in proinflammatory mediator, TNF-α released from KCs (**Fig. 1K**). Collectively, these data suggest that miR-146a expression is decreased in murine KCs following hepatic I/R, whereas IRAK1 and TRAF6 are increased.

### Downregulation of TRAF6 and IRAK1 by miR-146a in Macrophages Following Hypoxia/Reoxygenation

**Figure 2:**
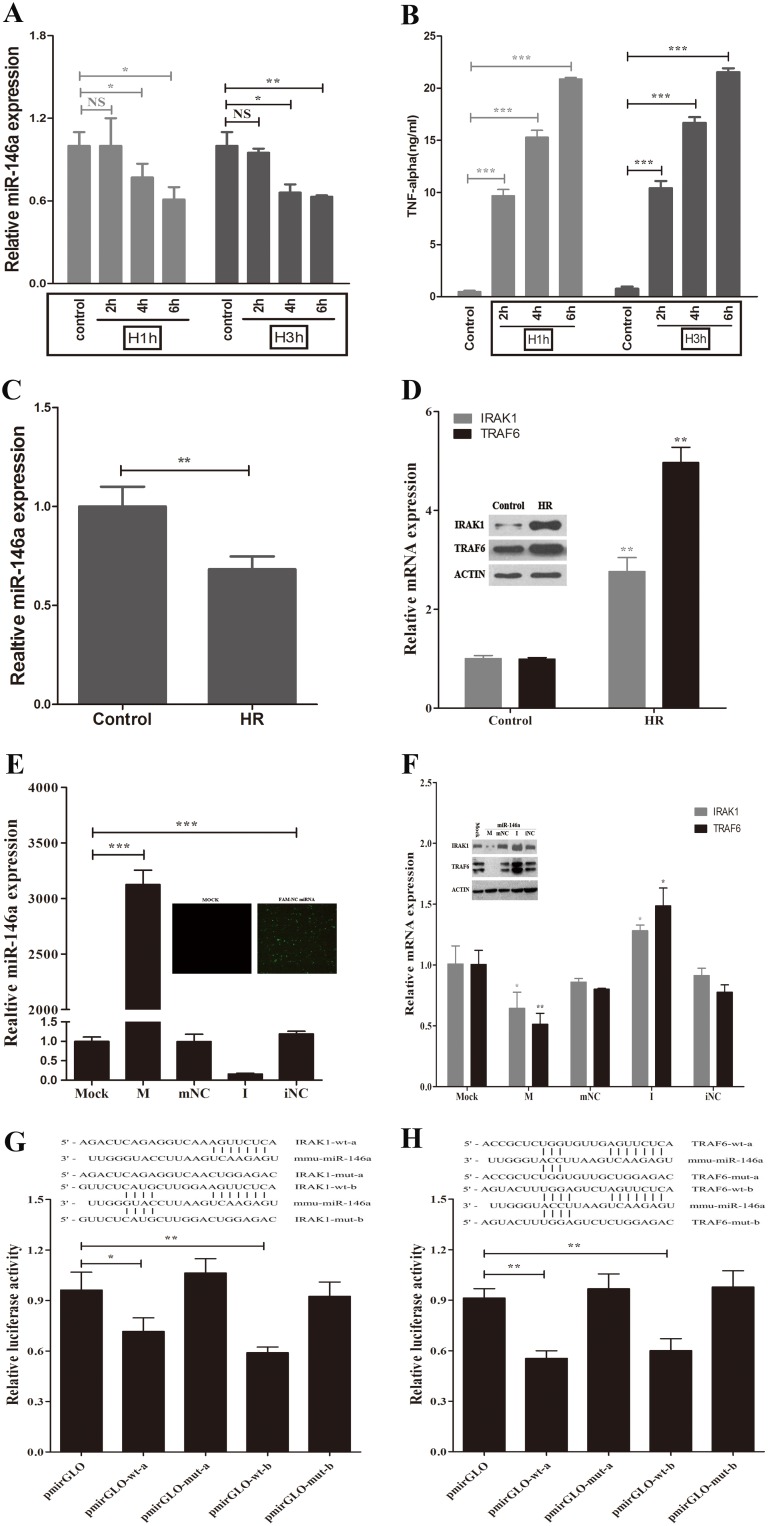
miR-146a directly downregulates IRAK1 and TRAF6 in macrophages after hypoxia/reoxygenation *in* *vitro*. RAW264.7 cells were exposed to 1 or 3 h of hypoxia and followed by various periods (2, 4, or 6 h) of reoxygenation. (A) qPCR analysis of miR-146a expression. (B) Supernatants were collected to measure TNF-α by ELISA. (C) The levels of miR-146a were decreased in macrophages following 3 h of hypoxia and 6 h of reoxygenation. (D) qRT-PCR analysis and immunoblotting of IRAK1 and TRAF6 in macrophages after hypoxia/reoxygenation (H/R). RAW 264.7 cells were transfected with miR-146a mimic, miR-146a inhibitor or relative control. (E) The efficacy of transfection was determined by qRT-PCR. (F) The mRNA and protein expression of IRAK1 and TRAF6 were determined by qRT-PCR and Western blot, respectively. (G and H) The sequence alignment of miR-146a and its putative and mutated target sites of the mouse IRAK1 and TRAF6 mRNA 3′untranslated region (3′UTR) were shown. RAW 264.7 cells were transiently co-transfected with luciferase report vectors, and in the presence of miR-146a mimic. Luciferase activities were normalized to the activity of Renilla luciferase. Results are mean ± SD. *, P<0.05 and **,P<0.01,***,P<0.001 versus negative control. H/R, hypoxia/reoxygenation; M, miR-146a mimic; mNC, negative control miRNA of miR-146a mimic; I, miR-146a inhibitor; iNC, negative control miRNA of miR-146a inhibitor.

To assay the effect of miR-146a on IRAK1 and TRAF6 expression *in* *vitro*, the well-established *in* *vitro* model of I/R, hypoxia/reoxygenation (H/R) model was applied [22], [23] in a murine macrophage cell line, RAW 264.7. RAW264.7 cells were exposed to 1 or 3 h of hypoxia (5% O_2)_ followed by various periods (2, 4, or 6 h) of reoxygenation (21% O_2)_. As shown in **Fig. 2A and B**, the production of inflammatory cytokines gradually increased after reoxygenation for 2–6 h, which was inversely associated with the reduction of miR-146a levels. We chose 3 h of hypoxia and 6 h of reoxygenation as the parameters for further experiments. RT-PCR and Western analysis revealed that TRAF6 and IRAK1 significantly increased in RAW264.7 cells following H/R (**Fig. 2D**). However, the levels of miR-146a decreased compared to cells cultured in 21% O_2_ (**Fig. 2C**). Overall, our data suggest that RAW264.7 cells subjected to H/R exhibited decreased miR-146a expression, elevated levels of TRAF6 and IRAK1, and the release of proinflammatory cytokines.

To determine the role of miR-146a on the expression of TRAF6 and TRAK1, we transfected miR-146a mimic, miR-146a inhibitor, or relative controls into RAW264.7 cells. RT-PCR analysis was first used to examine the efficacy of transfection. The miR-146a mimic significantly increased miR-146a levels, whereas the miR-146a inhibitor reduced endogenous miR-146a by 70% in RAW264.7 cells compared to the control miRNA (**Fig. 2E**). RAW264.7 cells were also transfected with a FAM-labeled negative control to observe the efficiency of transfection under inverted fluorescence microscopy (**Fig. 2E**). RT-PCR and Western analysis showed that the overexpression of miR-146a inhibited TRAF6 mRNA and protein levels in macrophages after H/R (**Fig. 2F**). Similar changes were observed for IRAK1 expression (**Fig. 2F**). In contrast, a decrease in miR-146a by the miR-146a inhibitor elevated TRAF6 and IRAK1 expression (**Fig. 2F**).

To further determine whether miR-146a inhibits TRAF6 and IRAK1 expression by directly binding to the 3′UTR of TRAF6 or IRAK1, we first used the miRNA target prediction software, Target Scan 5.2, to identify putative miR-146a binding sequences in the 3′UTR of TRAF6 and IRAK1 (**Fig. 2G and H**). A fragment of the 3′UTR of TRAF6 or IRAK1 containing the wild type (or mutated type) of miR-146a binding sequence was cloned into pmirGLO, a firefly luciferase reporter construct, and transfected into RAW264.7 cells with either the miR-146a mimic or its control. As expected, our data revealed that the miR-146a mimic inhibits luciferase activity by almost 50% (**Fig. 2G and H**). These results imply that miR-146 is able to directly bind to TRAF6 or IRAK1 regulatory sequences in their 3′UTRs, decreasing their expression in H/R-induced macrophages.

### miR-146a Reduces the Inflammatory Responses in Macrophages

**Figure 3:**
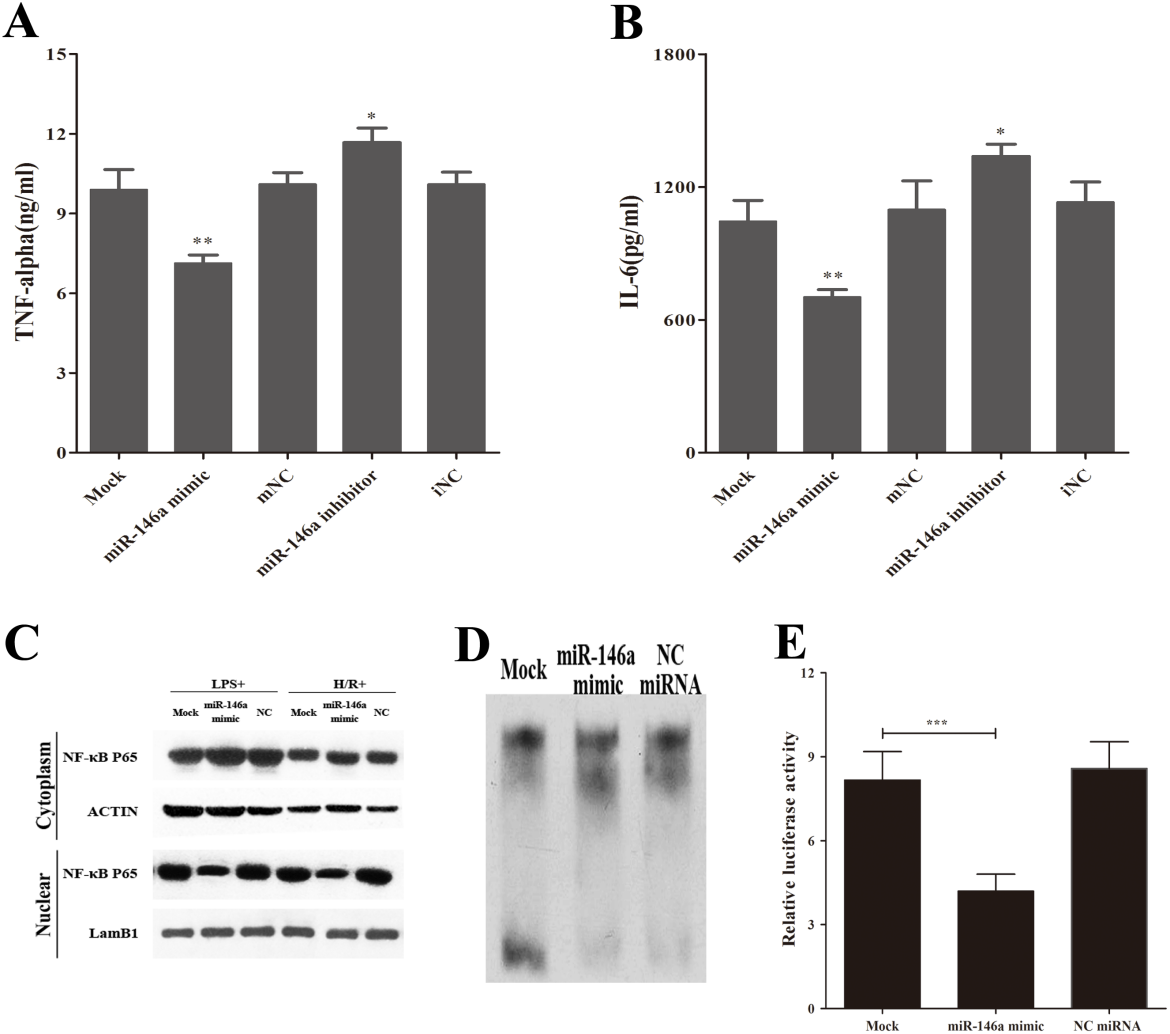
miR-146a attenuates NF-κB activation and reduces inflammatory responses in macrophages. RAW264.7 cells were transfected with miR-146a mimic and negative control miRNA for 24 h and followed hypoxia/reoxygenation. (A and B) Supernatants were collected to measure TNF-α and IL-6 by ELISA. (C) Cytosolic and nuclear p65 protein were separated using hypotonic buffer. β-actin and lamB1 indicate cytosolic and nuclear fraction, respectively. (D and E) EMSA and luciferase report assay of nuclear P65 activity. Co-culture of RAW264.7 cells/Kupffer cells with hepatocytes and followed H/R. Results are mean ± SD. *, P<0.05 and **,P<0.01,***,P<0.001 versus negative control. H/R, hypoxia/reoxygenation; mNC, negative control miRNA of miR-146a mimic; iNC, negative control miRNA of miR-146a inhibitor.

Activation of the TLR signaling pathway triggers NF-κB nuclear translocation through the recruitment of IRAK and TRAF family members, initiating an inflammatory cascade via the production of proinflammatory cytokines [8]. To elucidate the role of miR-146a on NF-κB activation and the production of proinflammatory mediators, transfected RAW 264.7 cells were examined after H/R. Our data showed that increased miR-146a attenuated the production of the proinflammatory cytokines, TNF-α and IL-6 in H/R- induced macrophages (**Fig. 3A and B**).

In addition, we examined NF-κB P65 localization in RAW264.7 cells and found that miR-146a inhibited the nuclear translocation of P65 after exposure to LPS or H/R (**Fig. 3C**). The luciferase reporter and EMSA were used to further confirm the role of miR-146a on the inactivation of nuclear P65. Under H/R exposure, the decreased level of NF-κB P65 activity in macrophages overexpressing exogenous miR-146a was observed (**Fig. 3D and E**). Overall, these data show that miR-146a attenuates the release of proinflammatory cytokines by inactivating transcription factors and NF-κB in LPS- or H/R-induced macrophages.

### TRAF6 and IRAK1 Mediate H/R-Induced Inflammatory Responses in Macrophages

**Figure 4:**
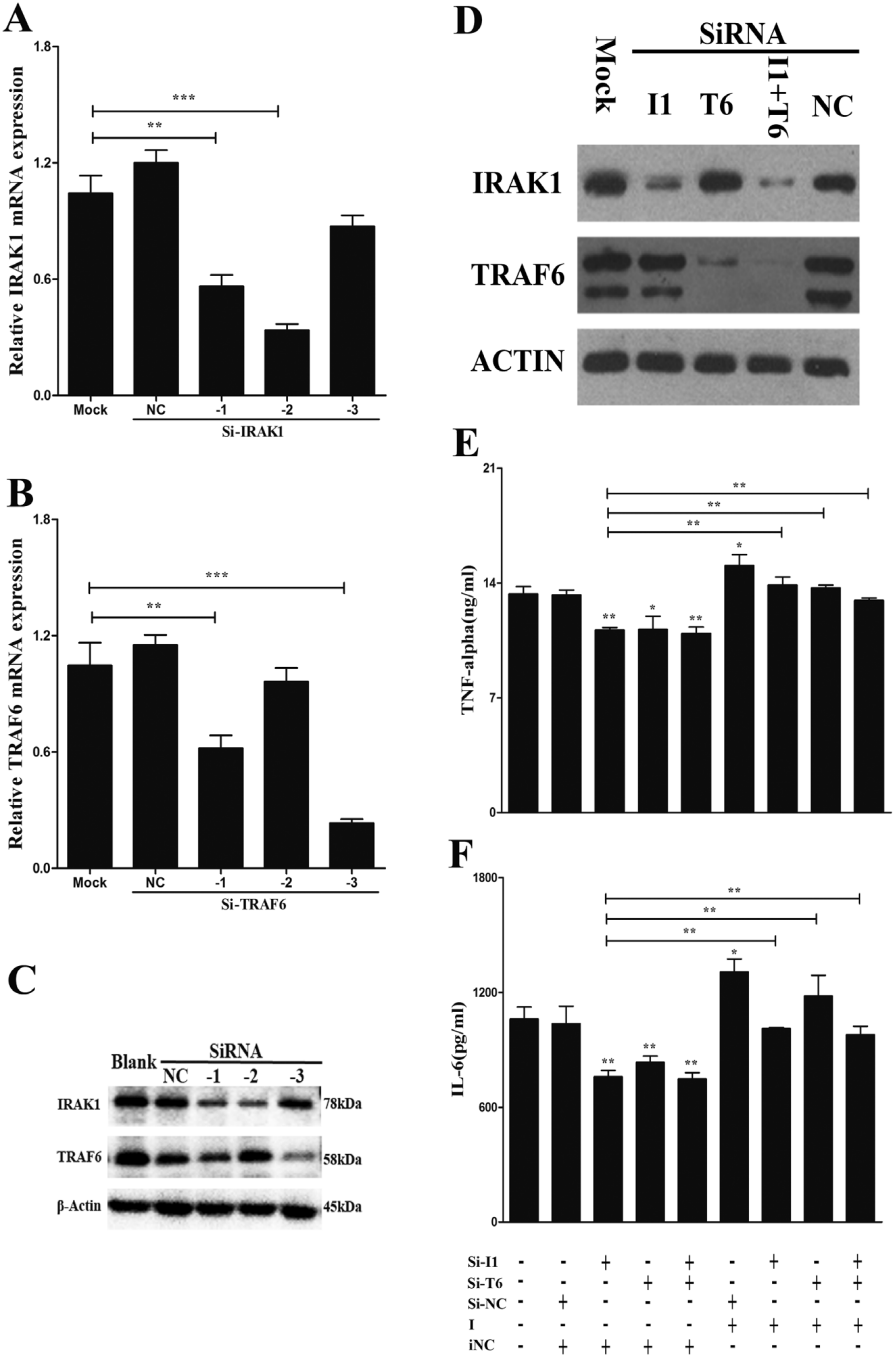
TRAF6 and IRAK1 decrease hypoxia/reoxygenation-induced inflammatory responses in macrophages. RAW264.7 cells were transfected with three siRNAs targeting IRAK1 or TRAF6 for 24(A and B) qRT-PCR analysis of IRAK1 and TRAF6. (C) Immunoblotting of IRAK1 and TRAF6. (D) Immunoblotting of IRAK1 and TRAF6 after transfected with the effective siRNA, negative control for 24 h. RAW264.7 cells were transfected with siRNA, negative control in presence of miR-146a inhibitor or its control for 24 h, and followed hypoxia/reoxygenation. (E and F) ELISA of TNF-α and IL-6 in supermatants. Results are mean ± SD. *, P<0.05 and **,P<0.01,***,P<0.001 versus negative control. I1, IRAK1; T6, TRAF6; NC, negative control siRNA; I, miR-146a inhibitor; iNC, negative control miRNA of miR-146a inhibitor.

To verify the function of TRAF6 and IRAK1 in miR-146a-mediated inflammatory response, we examined the role of TRAF6 and IRAK1 in H/R-induced proinflammatory cytokine production using RNA interference. The RNA interference efficacy of siRNAs were verified; an siRNA targeting IRAK1 and an siRNA targeting TRAF6 obviously decreased the corresponding mRNA and protein expression (**Fig. 4A–D**). An ELISA assay was used to determine the production of TNF-α and IL-6 released from RAW 264.7 cells co-transfected with these siRNAs, miR-146a inhibitor, or relative controls after H/R. Our data revealed that IRAK1 and TRAF6 silencing decreased the levels of proinflammatory cytokines (**Fig. 4E and F**). Interestingly, co-transfections with the miR-146a inhibitor abolished the reduction of TNF-α and IL-6 release compared to co-transfection with the siRNA and negative control miRNA (**Fig. 4E and F**). These results suggest that the specific suppression of IRAK1 and TRAF6 by miR-146a can reduce H/R-induced proinflammatory cytokine production.

### Effect of macrophages and miR-146a on hepatocyte apoptosis after H/R

**Figure 5:**
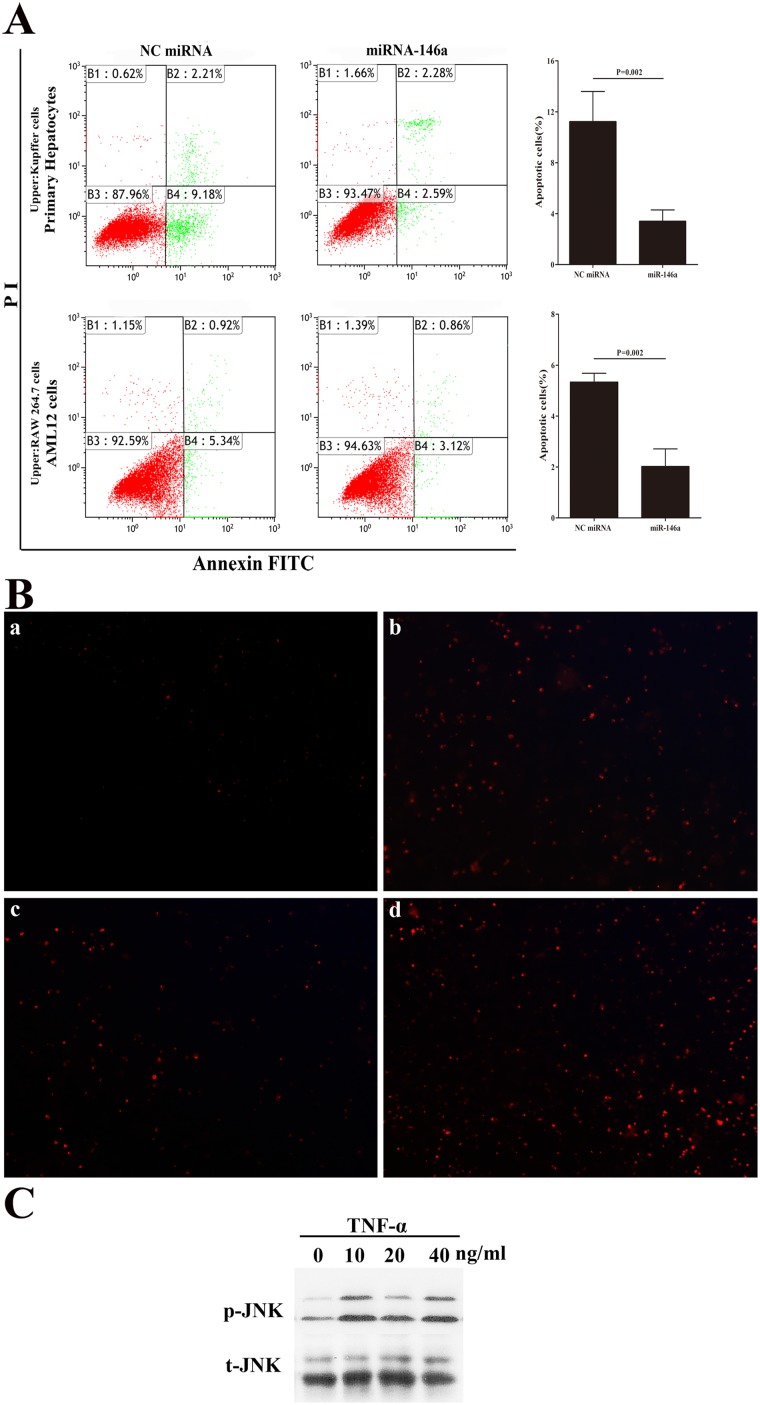
miR-146a reduce hepatocellular apoptosis. (A) Left panel. Representive FACS of hepatocellular apoptosis. Right panel. The percent of apoptotic cells were measured in three separate experiments. (B) The ROS levels were analyzed using DHE red fluorescence probe in AML12 cells stimulated with 0 (a), 10 ng/ml (b), 20 ng/ml (c), 40 ng/ml (d) TNF-α for 6 h. (C) Immunoblotting of phosphorylated and total JNK. Results are mean ± SD. Results are mean ± SD. *, P<0.05 and **,P<0.01,***,P<0.001 versus negative control.

To investigate potential interactions between macrophages and hepatocytes, an *in* *vitro* model imitating the liver microenvironment was used to assess whether the reduction of miR-146a in macrophages could decrease hepatocyte apoptosis during H/R injury. RAW264.7 cells, transfected with a miR-146a mimic or control, and mouse hepatocyte cell line, AML12 cells were cultured together in a trans-well system overnight and subsequently exposed to either normoxia or H/R (3 h/12 h). Using FACS analysis, mixed cultures of RAW 264.7 cells (transfected with the miR-146a mimic) and AML12 cells showed less hepatocyte apoptosis after H/R compared with control groups (**Fig. 5**). Interestingly, only AML12 cells exposed to H/R showed little hepatocyte apoptosis (data not shown). Next, KCs and hepatocytes were isolated from the same mice livers, and the co-culture of primary KCs and hepatocytes performed after transfecting the miR-146a mimic or negative control miRNA into KCs for 24 h. As shown in **Fig. 5**, the overexpression of miR-146a in KCs suppressed primary hepatocyte apoptosis following H/R. To confirm whether TNF-α induces the apoptosis of hepatocytes, we treated AML12 cells with 0, 10, 20, 40 ng/ml TNF-α alone for 6 h. As shown in **Fig. 5B**, we found that ROS was increased in AML12 cells stimulating with TNF-α. Moreover, the JNK protein also activated in treated AML12 cells (**Fig. 5C**). These data suggest that miR-146a may decrease hepatocyte injury after H/R through its effects on TNF-α secreting from macrophages.

### miR-146a attenuates the TLR signaling pathway during I/R Injury in the murine liver

**Figure 6:**
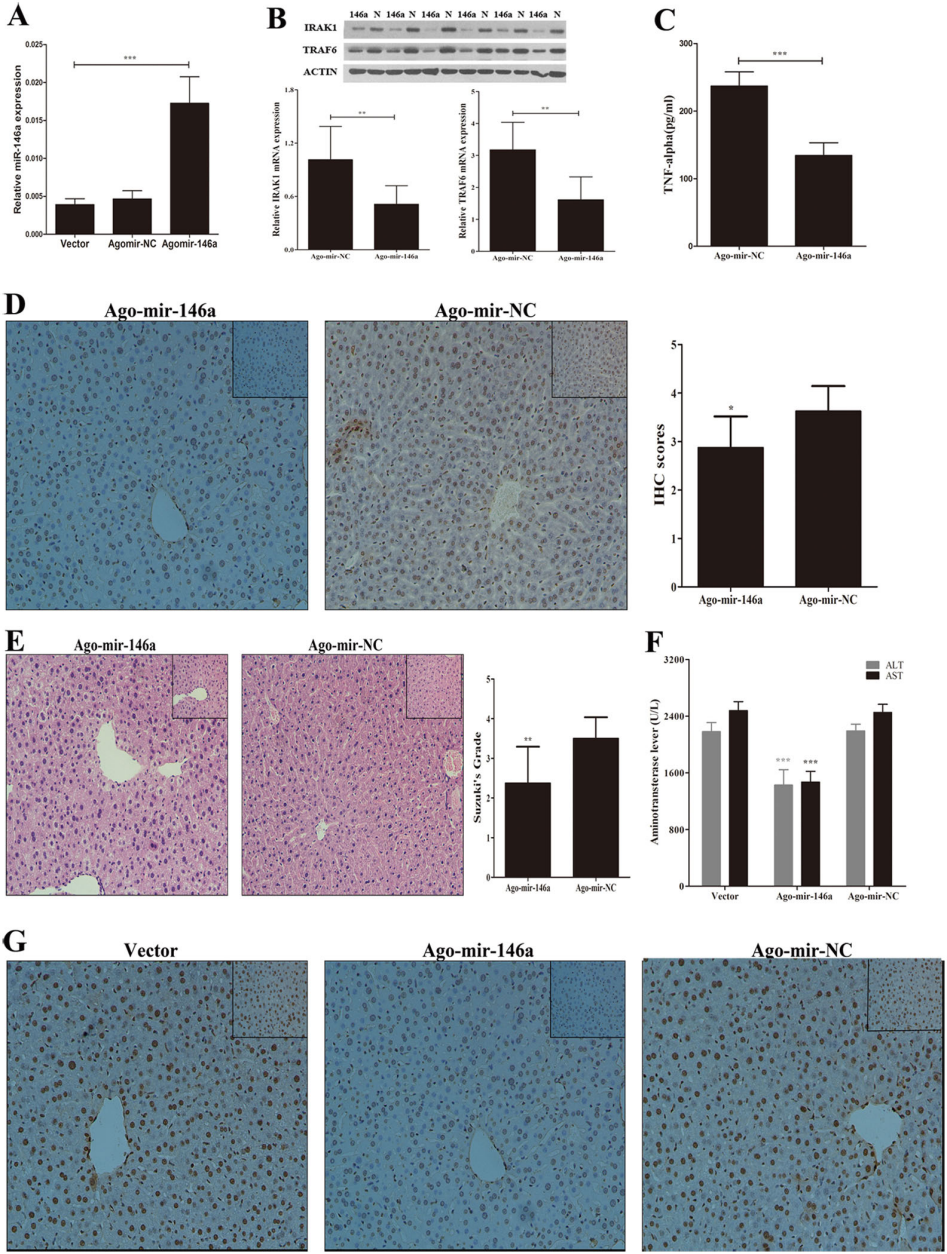
Transfection of Ago-miR-146a reduces liver ischemia/reperfusion injury *in* *vivo*. (A) Male mice (n = 3–4 mice per group) were treated with Ago-mir-146a, its control or vector for 24 h, qPCR analysis of mature miR-146a. With the same transfection, mice (n = 8–10 mice per group) were then subjected to I/R. (B) The mRNA and protein levels of IRAK1 and TRAF6 were determined by qPCR and Western Blot, respectively. (C) serum TNF-α were determined by ELISA. (D) Representive Immunohistochemistry staining and IHC scores of liver tissue for NF-κB p65 (magnification 200×). (E) Representive H&E staining (magnification ×200 and ×400 (inset)) and Suzuki’s grades. (F) serum ALT and AST. (G) TUNEL staining of hepatocellular apoptosis (magnification ×200 and ×400 (inset)). Results are mean ± SD. *, P<0.05 and **,P<0.01,***,P<0.001 versus negative control.

To verify our *in* *vitro* findings, male mice were treated with saline, Ago-mir-146a (a chemically-modified miR-146a), and negative control Ago-miRNA and subjected to I/R. Using RT-PCR analysis, we measured miR-146a expression in murine livers and found that mice subjected to treatment with Agomir-146a exhibited significant increases in miR-146a compared with negative control Ago-miRNA-treated mice (**Fig. 6A**). The overexpression of miR-146a led to the reduction of TRAF6 and IRAK1 expression after I/R (**Fig. 6B**). Meanwhile, the serum levels of TNF-α and positive nuclear expression of NF-κB P65 in cells decreased in Ago-miR-146a-treated mice following I/R compared to control groups (**Fig. 6C and D**). To elucidate the role of miR-146a on hepatocyte necrosis after I/R, levels of serum ALT and AST were tested in treated mice following liver I/R. As shown in **Fig. 6F**, the ALT and AST release in Ago-mir-146a-treated groups significantly decreased after reperfusion. H&E and TUNEL staining were further utilized to evaluate the severity of liver I/R injury. The alleviation of hepatocyte necrosis and a decrease in TUNEL-positive hepatic cells were found in mice upregulating miR-146a after hepatic I/R (**Fig. 6E and G**). Overall, our results indicate that miR-146a therapy could provide protection of the murine liver from injury after I/R via inactivation of the TLR signaling pathway.

## Discussion

Hepatic ischemia-reperfusion (I/R) injury often occurs during liver transplantation and liver surgery, impacting the outcome of patients. The pathophysiology of warm liver I/R injury involves the activation of the innate immune system, release of ROS, and hepatocellular injury [6], [24], [25]. The activation of a critical innate immune receptor, TLR4, has been established in hepatic I/R injury and TLR4 deficiency protects against liver I/R injury [7], [26]. The activation of TLR4 signaling on liver nonparenchymal cells, especially KCs, contributes to liver I/R injury. In addition, chimeric mice adoptively transferred with TLR4-mutant bone marrow cells are protected from hepatic I/R injury via inactivation of NF-κB and the reduction of proinflammatory cytokine production [27], [28].

Some miRNAs have been reported to take part in the development and function of immune cells, such as macrophages, monocytes, and natural killer cells. Specifically, miR-146a has recently been found to play an important role in the regulation of TLR4 signaling in macrophages [29]. We thus hypothesized that miR-146a may attenuate liver I/R injury through negatively regulating the TLR signaling pathway in KCs. This is the first study to find that the overexpression of exogenous miR-146a protects the mice liver from I/R injury. We have demonstrated that the suppression of NF-κB activation and a decrease in the release of proinflammatory mediators, which may improve effects from hepatic I/R injury, occurs via IRAK1 and TRAF6 expression mediated by mir-146a in macrophages.

It is well established that miRNAs play an important role in several animal models of I/R injury [10], [30]. In addition, our previous research has suggested that miR-146a expression is decreased, and IRAK1 and TRAF6 expression upregulated, during liver I/R injury [14]. We thus postulate that hepatic I/R induces liver injury via reduction of miR-146a targeting the key kinases of TLR signaling. Indeed, we have found that levels of miR-146a are decreased after 8 h reperfusion following 60 min ischemia, whereas the levels of IRAK1 and TRAF6 are elevated and the severity of hepatocellular injury is poorer. In recent reports, miR-146a expression induced by activation of TLR signaling is NF-κB-dependent in human and mouse macrophages [13], [31]. Activation of KCs in the early period of I/R injury may induce NF-κB nuclear translocation and binding to the promoter of miR-146a, subsequently leading to mature miR-146a expression. This may explain why in the early phase of reperfusion (∼2 h) miR-146a is significantly increased. When we examined the expression of miR-146a in KCs and hepatocytes isolated from mice livers after I/R, we found that miR-146a is decreased in KCs with the upregulation of TRAF6 and IRAK1 expression; however, the levels of miR-146a remained unchanged in hepatocytes. We also obtained changes similar to KCs following liver I/R in mouse macrophages (RAW 264.7 cells) exposed to 3 h of hypoxia and 6 h of reoxygenation.

To test the role of miR-146a in the TLR signaling pathway, we transfected macrophages with miR-146a mimic or negative control miRNA and found that transfection of miR-146a significantly suppresses IRAK1 and TRAF6 expression, likely interfering with expression through the 3′UTR. Similarly, the *in* *vivo* overexpression of miR-146a by transfection with Ago-miR-146a decreased the levels of IRAK1 and TRAF6, leading to the inactivation of NF-κB. Our observation is consistent with recent reports showing that miR-146a targets IRAK1 and TRAF6 and negatively regulates the TLR signaling pathway [10], [30], [32]. Moreover, miR-146a–null mice revealed a marked increase in TRAF6 and IRAK1 protein levels in BM-derived macrophages in comparison with WT mice [33]. However, whether miR-146a knockout mice are more sensitive to liver I/R injury is needed to be further confirmed. In addition, our data show that the suppression of IRAK1 and TRAF6 by miR-146a results in the inhibition of NF-κB nuclear translocation and binding activity and reduces the release of proinflammatory mediators.

IRAK1 and TRAF6 are two key cytoplasmic protein kinases induced by I/R via TLR signaling. Phosphorylation of these kinases results in the nuclear translocation of the transcription factor NF-κB, which results in the production of proinflammatory cytokines, including TNF-α and IL-6 [7]–[9]. We found that knockdown of IRAK1 or/and TRAF6 significantly reduce the release of inflammatory mediators in macrophages following H/R *in* *vitro*. Considerable research has shown that TLR4-mediated inflammatory responses are impaired in IRAK1-deficient or TRAF6-deficient macrophages [34], [35]. A recent report showed that IRAK1-deficient mice were protected from intestinal I/R-mediated tissue damage with a reduction of proinflammatory cytokines secretion [10]. In addition, when we co-transfected macrophages with a miR-146a inhibitor and siRNA, it abolished the reduction of TNF-α and IL-6 release. These data suggest that IRAK1 and TRAF6 may be important targets of miR-146 in the reduction of proinflammatory cytokines release.

We also observed that transfection of Ago-miR-146a *in* *vivo* significantly decreases hepatocellular apoptosis and improves hepatic function following liver I/R. The precise mechanism of the protection effect in the liver microenvironment during liver I/R is elusive. Furthermore, our *in* *vitro* co-culture of hepatocytes and KCs/macrophages indicated that H/R caused hepatocellular apoptosis; however, with KCs/macrophages transfected with miR-146a, the FITC-positive hepatocytes (indicating apoptosis) were obviously reduced. Interestingly, when hepatocytes alone were exposed to H/R little apoptosis was observed. To further examine the role of TNF-α on hepatocytes, we stimulated AML12 cells with TNF-α for 6 h. We found the increase of ROS and activation of JNK in treated AML12 cells. ROS and activated JNK can induce the apoptosis of hepatocytes [35]. These data indicate that overexpression of miR-146a in macrophages negatively regulates the TLR signaling pathway and decreases the production of TNF-α, may indirectly reduce the apoptosis of hepatocytes. The TLR signaling pathway activates different intrahepatic cell types, including dendritic cells (DCs) [36], sinusoidal endothelial cells (SECs) [37] and hepatocytes [38], and we cannot exclude the effects of miR-146a on these other cell types. It is possible that the overexpression of miR-146a in DCs or SECs further regulates I/R injury. We and other authors previously reported that regulatory T cells (Tregs) contribute to protective effects during I/R injury [39]–[41]. In addition, Lu et al.[42] reported that miR-146a plays an important role in Treg-mediated control of Th1 response. These results indicated that miR-146a may attenuate liver reperfusion injury through additional pathways and this requires further investigation.

In summary, we have demonstrated that therapeutic approaches using miR-146a could ameliorate liver I/R injury in the warm segmental liver I/R model *in* *vivo* and H/R model *in* *vitro*. The mechanism of protection involves the inactivation of NF-κB and the reduction of proinflammatory cytokine production through the direct suppression of IRAK1 and TRAF6. Furthermore, recent reports have found that natural diindolylmethase [10] or VELCADE [43] could upregulate miR-146a and minimize I/R injury. These results suggest that therapeutic modulation of the TLR signaling pathway and innate immune system in the liver, by the administration of miR-146a or its inducers, is a promising goal for protecting the liver from I/R injury.

## Supporting Information

Table S1 **Sequences of real-time PCR primers.**
(DOC)

Table S2 **Oligonucleotide sequences for small RNA.**
(DOC)

Table S3 **Oligonucleotide sequences (wild type and mutated).**
(DOC)
