# Correction: miR-146a Ameliorates Liver Ischemia/Reperfusion Injury by Suppressing IRAK1 and TRAF6

**DOI:** 10.1371/journal.pone.0288672

**Published:** 2023-07-11

**Authors:** Weiwei Jiang, Liangliang Kong, Qingfeng Ni, Yeting Lu, Wenzhou Ding, Guoqing Liu, Liyong Pu, Weibing Tang, Lianbao Kong

Following the publication of this article [1], concerns were raised regarding results presented in Fig 6. Specifically, the Fig 6D Ago-mir-146a panel appeared similar to the Fig 6G Ago-mir-146a panel despite being used to represent different experimental conditions.

The corresponding author explained that during the initial rounds of revision of this manuscript the incorrect images were accidentally used to prepare the Fig 6D panel. They provided an updated Fig 6 with the correct Fig 6D Ago-mir-146a image obtained in the original experiment, as well as the individual level data underlying the published results (S1 File).

Editorial review of these data raised concerns that the underlying data presented for Fig 6C presents 10 Ago-mir-NC samples and 8 Ago-mir-146a samples, whereas the underlying data for Figs 6D, 6E, and 6F all present 8 Ago-mir-NC samples and 10 Ago-mir-146a samples. The corresponding author explained that 10 mice were used in each group whilst performing liver ischemia/reperfusion experiments, but that two liver samples of the Ago-mir-NC group were damaged during embedding or sectioning, and as a result this group only contained 8 samples. The Fig 6C results present ELISA data obtained from serum samples; authors intended to match sample sizes for this experiment and Figs 6D–6F, but instead conducted the Fig 6C experiment with n = 8 samples in the Ago-mir-146a and n = 10 samples in the Ago-mir-NC group. The corresponding author stated that the data shown in the figure are correctly labelled as to the experimental groups.

**Fig 6:**
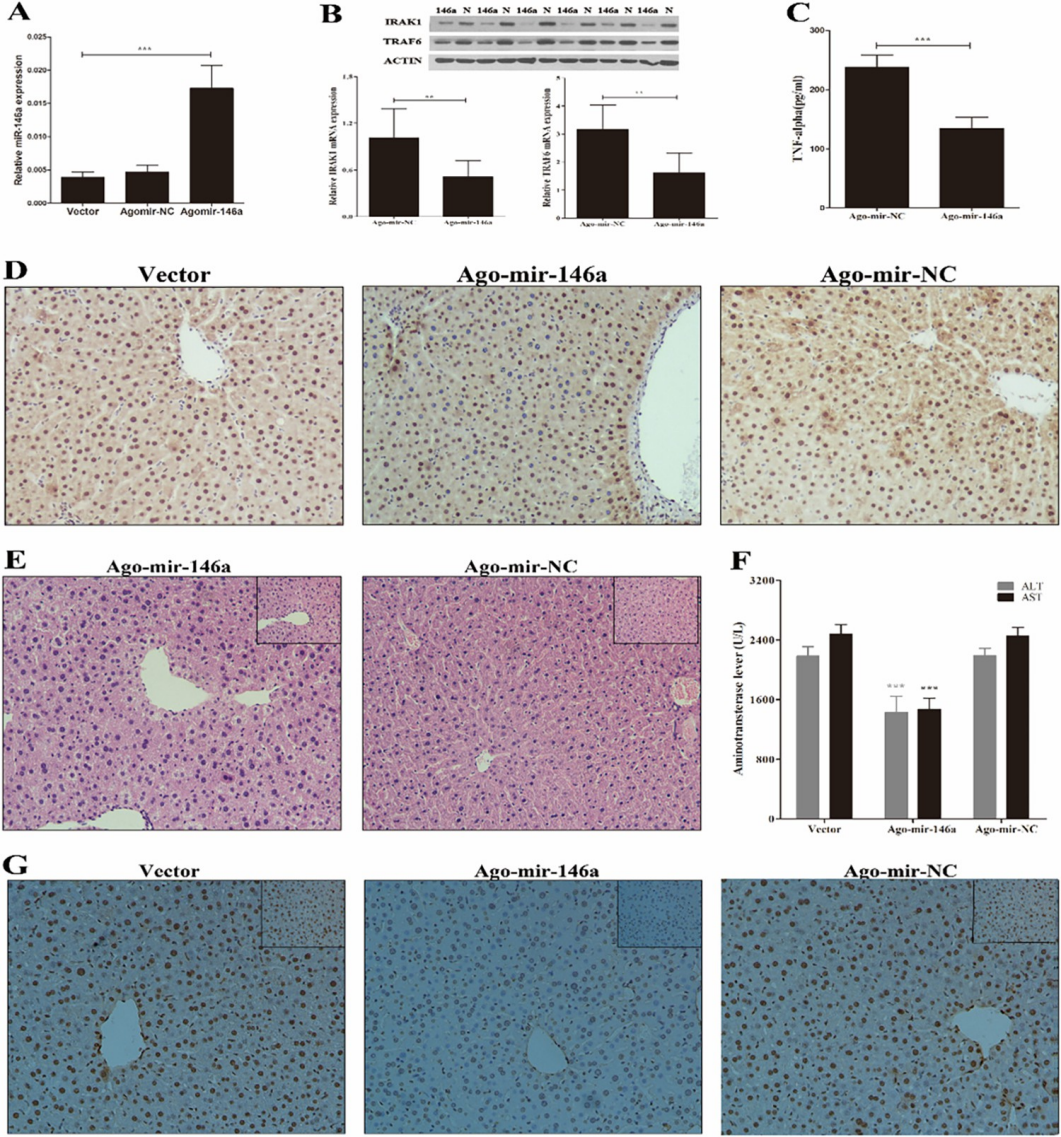
Transfection of Ago-miR-146a reduces liver ischemia/reperfusion injury *in vivo*. (A) Male mice (n  =  3–4 mice per group) were treated with Ago-mir-146a, its control or vector for 24 h, qPCR analysis of mature miR-146a. With the same transfection, mice (n  =  8–10 mice per group) were then subjected to I/R. (B) The mRNA and protein levels of IRAK1 and TRAF6 were determined by qPCR and Western Blot, respectively. (C) serum TNF-α were determined by ELISA. (D) Representive Immunohistochemistry staining and IHC scores of liver tissue for NF-κB p65 (magnification 200×). (E) Representive H&E staining (magnification ×200 and ×400 (inset)) and Suzuki’s grades. (F) serum ALT and AST. (G) TUNEL staining of hepatocellular apoptosis (magnification ×200 and ×400 (inset)). Results are mean ± SD. *, P<0.05 and **,P<0.01,***,P<0.001 versus negative control.

## Supporting information

S1 File Individual level data underlying the Fig 6A–6G results. (ZIP)

S2 File Original western blot data underlying the Fig 6B results. (ZIP)
