# Supplementary material for: miR-146a Ameliorates Liver Ischemia/Reperfusion Injury by Suppressing IRAK1 and TRAF6
Source: PLoS One. 2014 Jul 2;9(7):e101530. doi: 10.1371/journal.pone.0101530 (PMC4079695; doi:10.1371/journal.pone.0101530)
Supplement: Table S1 — Sequences of real-time PCR primers. (DOC) [file pone.0101530.s001.doc]

**Supplementary table1:Sequences of real-time PCR primers**

| **Gene name** | **Gene ID** |  | **Sequences**  Reverse primer | **Size(bp)** |
| --- | --- | --- | --- | --- |
| IRAK1 | 16179 | **Sense:** | *5'-CCTGGGTTATGTGCCGCTT-3'* | 191 |
|  |  | **Anti-sense:** | *5'-GAGGATGTGAACGAGGTCAGC-3'* |  |
| TRAF6 | 22034 | **Sense:** | *5'-ATGCAGAGGAATCACTTGGCA-3'* | 103 |
|  |  | **Anti-sense:** | *5'ACGGACGCAAAGCAAGGTT-3'* |  |
| beta-actin | 11461 | **Sense:** | *5'-GTGACGTTGACATCCGTAAAGA-3'* | 245 |
|  |  | **Anti-sense:** | *5'-GCCGGACTCATCGTACTCC-3'* |  |
| **Gene name** | **ABI probe ID** | **Sequences** | | **Size(bp)** |
| mmu-mir-146a | 000468 | *UGAGAACUGAAUUCCAUGGGUU* | | 22 |
| mmu-SNRU6 | 001973 |  | |  |
