# Supplementary material for: miR-146a Ameliorates Liver Ischemia/Reperfusion Injury by Suppressing IRAK1 and TRAF6
Source: PLoS One. 2014 Jul 2;9(7):e101530. doi: 10.1371/journal.pone.0101530 (PMC4079695; doi:10.1371/journal.pone.0101530)
Supplement: Table S2 — Oligonucleotide sequences for small RNA. (DOC) [file pone.0101530.s002.doc]

**Supplementary table2:Oligonucleotide sequences for small RNA**

| **Name** | **No.** |  | **Sequences** |
| --- | --- | --- | --- |
| Si-IRAK1 | -1 | **Sense:** | CGAGCAGUCAUGAGAAAUATT |
|  |  | **Anti-sense:** | UAUUUCUCAUGACUGCUCGTT |
|  | -2 | **Sense:** | UAGAGUGGACUAUGGUGAATT |
|  |  | **Anti-sense:** | UUCACCAUAGUCCACUCUATT |
|  | -3 | **Sense:** | CUGCCCAGAUCUAUAAGAATT |
|  |  | **Anti-sense:** | UUCUUAUAGAUCUGGGCAGTT |
| Si-TRAF6 | -1 | **Sense:** | GCGCUGUGCAAACUAUAUATT |
|  |  | **Anti-sense:** | UAUAUAGUUUGCACAGCGCTT |
|  | -2 | **Sense:** | CCCAGGCUGUUCAUAAUGUTT |
|  |  | **Anti-sense:** | ACAUUAUGAACAGCCUGGGTT |
|  | -3 | **Sense:** | GCUACGAUGUGGAGUUUGATT |
|  |  | **Anti-sense:** | UCAAACUCCACAUCGUAGCTT |
| Si-NC | -1 | **Sense:** | UUCUCCGAACGUGUCACGUTT |
|  |  | **Anti-sense:** | ACGUGACACGUUCGGAGAATT |
| **Name** | |  | **Sequences** |
| mmu-miR-146a  mimics | | **Sense:** | UGAGAACUGAAUUCCAUGGGUU |
|  |  | **Anti-sense:** | CCCAUGGAAUUCAGUUCUCAUU |
| mmu-miR-146a inhibitors | | | AACCCAUGGAAUUCAGUUCUCA |
| mmu-miR-146a NC | | | CAGUACUUUUGUGUAGUACAA |
