# Supplementary material for: miR-146a Ameliorates Liver Ischemia/Reperfusion Injury by Suppressing IRAK1 and TRAF6
Source: PLoS One. 2014 Jul 2;9(7):e101530. doi: 10.1371/journal.pone.0101530 (PMC4079695; doi:10.1371/journal.pone.0101530)
Supplement: Table S3 — Oligonucleotide sequences (wild type and mutated). (DOC) [file pone.0101530.s003.doc]

**Supplementary table3:Oligonucleotide sequences(wild type and mutated)**

Oligonucleotide sequences(wild type)

| IRAK1-a | Sense: | *CTAGC*AGACTCAGAGGTCAA**AGTTCTCA***C* |
| --- | --- | --- |
|  | Anti-sense: | *TCGAG*TGAGAACTTTGACCTCTGAGTCT*G* |
| IRAK1-b | Sense: | *CTAGCG*TTCT**CATG**CTTGGA**AGTTCTCA***C* |
|  | Anti-sense: | *TCGAG*TGAGAACTTCCAAGCATGAGAAC*G* |
| TRAF6-a | Sense: | *CTAGC*ACCGCTC**TGG**TGTTG**AGTTCTC**A*C* |
|  | Anti-sense: | *TCGAG*TGAGAACTCAACACCAGAGCGGT*G* |
| TRAF6-b | Sense: | *CTAGC*AGTACTT**TGGA**GTCT**AGTTCTC**A*C* |
|  | Anti-sense: | *TCGAG*TGAGAACTAGACTCCAAAGTACT*G* |

Oligonucleotide sequences(mutated)

| IRAK1-a | Sense: | *CTAGC*AGACTCAGAGGTCAA**CTGGAGAC***C* |
| --- | --- | --- |
|  | Anti-sense: | *TCGAG*GTCTCCAGTTGACCTCTGAGTCT*G* |
| IRAK1-b | Sense: | *CTAGC*GTTCTCATGCTTGGA**CTGGAGAC***C* |
|  | Anti-sense: | *TCGAG*GTCTCCAGTCCAAGCATGAGAAC*G* |
| TRAF6-a | Sense: | *CTAGC*ACCGCTC**TGG**TGTTG**CTGGAGAC***C* |
|  | Anti-sense: | *TCGAG*GTCTCCAGCAACACCAGAGCGGT*G* |
| TRAF6-b | Sense: | *CTAGC*AGTACTT**TGGA**GTCT**CTGGAGAC***C* |
|  | Anti-sense: | *TCGAG*GTCTCCAGAGACTCCAAAGTACT*G* |

Italic and underline shows restriction enzymes *NheI(G^CTAGC)* and *XhoI(C^TCGAG)* site.**Bold** shows target sequences.
