# Supplementary figures and images for: Correction: miR-146a Ameliorates Liver Ischemia/Reperfusion Injury by Suppressing IRAK1 and TRAF6
Source: PLoS One. 2023 Jul 11;18(7):e0288672. doi: 10.1371/journal.pone.0288672 (PMC10335655; doi:10.1371/journal.pone.0288672)

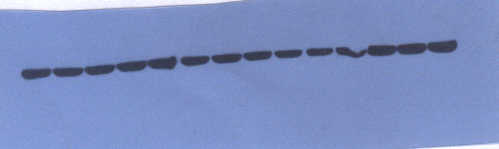

Supplement: S2 File — (ZIP) [file pone.0288672.s002.zip › S2 File/ACTIN.tif]

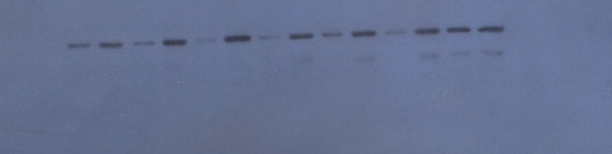

Supplement: S2 File — (ZIP) [file pone.0288672.s002.zip › S2 File/IRAK1.tif]

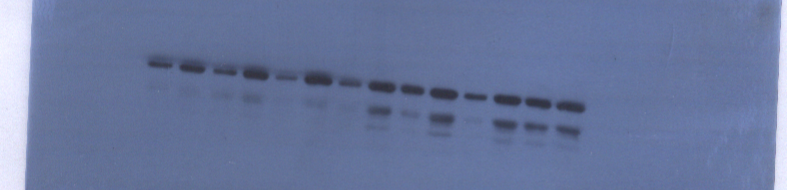

Supplement: S2 File — (ZIP) [file pone.0288672.s002.zip › S2 File/TRAF6.tif]
